# Whole-genome sequencing of Acinetobacter baumannii clinical isolates from a tertiary hospital in Terengganu, Malaysia (2011–2020), revealed the predominance of the Global Clone 2 lineage

**DOI:** 10.1099/mgen.0.001345

**Published:** 2025-02-05

**Authors:** Nurul Saidah Din, Farahiyah Mohd. Rani, Ahmed Ghazi Alattraqchi, Salwani Ismail, Nor Iza A. Rahman, David W. Cleary, Stuart C. Clarke, Chew Chieng Yeo

**Affiliations:** 1 Centre for Research in Infectious Diseases and Biotechnology, Faculty of Medicine, Universiti Sultan Zainal Abidin, Kuala Terengganu, Malaysia; 2 Department of Microbes, Infections and Microbiomes, School of Infection, Inflammation and Immunology, College of Medicine and Health, University of Birmingham, Birmingham, UK; 3 Institute of Microbiology and Infection, University of Birmingham, Birmingham, UK; 4 Faculty of Medicine and Institute for Life Sciences, University of Southampton, Southampton, UK; 5 NIHR Southampton Biomedical Research Centre, University Hospital Southampton Foundation NHS Trust, Southampton, UK; 6 Global Health Research Institute, University of Southampton, Southampton, UK; 7 Institute for Research, Development and Innovation, International Medical University, Kuala Lumpur, Malaysia

**Keywords:** *Acinetobacter baumannii*, GC2 lineage, Malaysia, multidrug resistant, genome sequencing

## Abstract

Carbapenem-resistant *Acinetobacter baumannii* is recognized by the World Health Organization (WHO) as one of the top priority pathogens. Despite its public health importance, genomic data of clinical isolates from Malaysia remain scarce. In this study, whole-genome sequencing was performed on 126 *A*. *baumannii* isolates collected from the main tertiary hospital in the state of Terengganu, Malaysia, over a 10-year period (2011–2020). Antimicrobial susceptibilities determined for 20 antibiotics belonging to 8 classes showed that 77.0% (*n*=97/126) of the isolates were categorized as multidrug resistant (MDR), with all MDR isolates being carbapenem resistant. Multilocus sequence typing analysis categorized the Terengganu *A. baumannii* clinical isolates into 34 Pasteur and 44 Oxford sequence types (STs), with ST2_Pasteur_ of the Global Clone 2 lineage identified as the dominant ST (*n*=76/126; 60.3%). The ST2_Pasteur_ isolates could be subdivided into six Oxford STs with the majority being ST195_Oxford_ (*n*=35) and ST208_Oxford_ (*n*=17). Various antimicrobial resistance genes were identified with the *bla*_OXA-23_-encoded carbapenemase being the predominant acquired carbapenemase gene (*n*=90/126; 71.4%). Plasmid-encoded *rep* genes were identified in nearly all (*n*=122/126; 96.8%) of the isolates with the majority being Rep_3 family (*n*=121). Various virulence factors were identified, highlighting the pathogenic nature of this bacterium. Only 14/126 (11.1%) of the isolates were positive for the carriage of CRISPR-Cas arrays with none of the prevalent ST2_Pasteur_ isolates harbouring them. This study provided a genomic snapshot of the *A. baumannii* isolates obtained from a single tertiary healthcare centre in Malaysia over a 10-year period and showed the predominance of a single closely related ST2_Pasteur_ lineage, indicating the entrenchment of this clone in the hospital.

**Impact Statement**

The bacterium *Acinetobacter baumannii* has been listed by the World Health Organization (WHO) as a critical priority antibiotic-resistant pathogen. Despite its importance, there is a scarcity of genomic data of *A. baumannii* from low- and middle-income countries like Malaysia. Here, we present the whole-genome analyses of 126 *A*. *baumannii* isolates from a single tertiary hospital in Malaysia obtained over a 10-year period (2011–2020) to address the current knowledge gap. We found the predominance of a single, closely related clonal lineage designated as Global Clone 2 (GC2), inferring the entrenchment of this clone in the hospital. Of concern, majority of the *A. baumannii* isolates were resistant to multiple antibiotics and most were resistant to carbapenems, which are the drugs of choice for the treatment of *A. baumannii* infections. Various antibiotic resistance genes were identified with *bla*_OXA-23_ being the most predominant gene mediating carbapenem resistance in the Malaysian isolates. Although all the GC2 *A. baumannii* isolates harboured *bla*_OXA-23_, the gene was also found in other varied lineages, indicating that there is also a continuous circulation of multiple different antibiotic-resistant *A. baumannii* clones in the Malaysian hospital.

## Data Summary

The authors confirm that all supporting data, code and protocols have been provided within the article or through supplementary data files.

## Introduction

*Acinetobacter baumannii* is an aerobic Gram-negative coccobacillus that has become a major nosocomial pathogen and a common isolate in the intensive care unit. Nosocomial infections are believed to be responsible for ~1.4 million infections every year, and *A. baumannii* has been reported as one of the major contributors [1]. Clinical manifestations of *A. baumannii* infections include skin or soft tissue infections, pneumonia, meningitis, urinary tract infection and bacteraemia [2,3]. * A. baumannii* infections, especially from multidrug- and carbapenem-resistant strains, were recorded to be substantially higher in Southeast Asia compared to other regions at 58.51 and 64.91%, respectively [4]. The prevalence of multidrug-resistant (MDR) *A. baumannii* infections in nosocomial pneumonia patients has been reported to be as high as 95% and linked to mortality rates of up to 85% [5].

The World Health Organization (WHO) has classified carbapenem-resistant *A. baumannii* as one of the top critical priority pathogens [6,7], a member of the so-called ‘ESKAPE’ group of pathogens along with five others that are in critical need of novel antibiotics, namely, *Enterococcus faecium*, *Staphylococcus aureus*, *Klebsiella pneumoniae*, *Pseudomonas aeruginosa* and *Enterobacter* spp. [8]. In Malaysia, the latest National Antibiotic Resistance Surveillance Report (NSAR) for the year 2022 recorded a slight decrease in the incidence of resistance in *A. baumannii* compared to the previous year (2021) for seven tested antibiotics, i.e. amikacin, ampicillin/sulbactam, cefoperazone/sulbactam, ceftazidime, gentamicin, imipenem and meropenem. Nevertheless, carbapenem resistance remained above 60%, whereas in 2018, carbapenem resistance was only at around 40% [9].

One of the features that enabled *A. baumannii* to gain clinical prominence in the last two decades is its remarkable ability to rapidly acquire and develop resistance to nearly all classes of antibiotics. The emergence of MDR *A. baumannii* is generally attributed to the capability of *A. baumannii* to exhibit multiple mechanisms of resistance including enzymatic degradation of antibiotics, modification of target sites, upregulation of efflux pumps and permeability alterations in the outer membrane [10,12]. The flexibility and adaptive feature of the *A. baumannii* genome lead to the accumulation of antibiotic-resistant determinants mainly through horizontal gene transfer [13].

Understanding the genomic characteristics of *A. baumannii* clinical isolates is one step towards establishing a better protocol to prevent the development of antibiotic resistance and improve the treatment management of patients. Despite the importance of *A. baumannii*, there have been scarce data on the genomes of Malaysian isolates. In this study, whole-genome sequencing was performed on a total of 126 isolates obtained from Hospital Sultanah Nur Zahirah (HSNZ), the main tertiary hospital in the state of Terengganu, Malaysia, throughout a 10-year period (2011–2020). This enabled us to gain important insights into the genetic lineages of *A. baumannii* that are found in the hospital as well as to compare with the genomes of other relevant *A. baumannii* isolates that are publicly available.

## Methods

### Bacterial collection

In this study, a total of 126 *A*. *baumannii* isolates collected from the Microbiology Unit of the Department of Pathology, HSNZ, throughout the years 2011 to 2020 (but without any strains from 2013 and 2014), were revived from stored frozen stock cultures. All isolates were obtained from clinical samples taken from non-duplicated patients. The isolates were primarily identified as *A. baumannii* via standard phenotypic biochemical methods at the hospital laboratory. Further reidentification was done by sequencing the RNA polymerase *β*-subunit-encoded gene, *rpoB* [14].

### Phenotypic antimicrobial susceptibility testing

The antimicrobial susceptibility testing (AST) was determined using the Kirby–Bauer disc diffusion method against 20 clinically relevant antibiotics from 8 classes of antimicrobials. In brief, a bacterial inoculum of 0.5 McFarland standard was dipped with a sterile cotton swab and streaked over the entire Mueller–Hinton agar (Oxoid, Basingstoke, UK) plate surface. The discs containing the antibiotics were placed onto the surface of the inoculated agar plate and incubated at 37 °C for 24 h. The zone of inhibition diameter results were interpreted according to the Clinical and Laboratory Standards Institute (CLSI) M100 guidelines [15] as either susceptible, intermediate resistant or resistant. The resistance profile was categorized as MDR when the isolate exhibited resistance to one or more antibiotics in three or more classes of antimicrobial agent [16].

### DNA extraction and whole-genome sequencing analysis

The genomic DNA of the 126 *A*. *baumannii* Terengganu clinical isolates were extracted using GeneJET Genomic DNA Purification Kit (Thermo Scientific™) following the manufacturer’s instruction for Gram-negative bacteria. The extracted DNA was visualized on 1% (w/v) agarose gel electrophoresis and was quantified with Implen NanoPhotometer® spectrophotometer (Implen, Munich, Germany). The *A. baumannii* Terengganu isolates collected from the years 2011–2016 were sequenced by commercial sequencing providers with a paired-end sequencing strategy using the Illumina HiSeq platform (HiSeq-PE150) (Novogene, Singapore), while the isolates collected from the years 2017–2020 were sequenced on the DNBSEQ platform (Beijing Genome Institute, Beijing, China). FastQC (https://www.bioinformatics.babraham.ac.uk/projects/fastqc/) [17] was used to check the quality of the obtained raw reads. The adaptor sequences, contamination and low-quality reads were filtered and removed.

The paired-end reads were *de novo* assembled using UniCycler v0.4.8 (https://github.com/rrwick/Unicycler) [18], and the quality of the resulting contigs was determined using QUAST v5.2.0 (https://github.com/ablab/quast) [19]. The assembled genomes were annotated using PROKKA (https://github.com/tseemann/prokka), and the generated General Feature Format (GFF) file was then subjected to Roary (https://github.com/sanger-pathogens/Roary) to obtain the core-genome alignment using the criteria of aa sequence identities of 95% and presence in 99% of genomes [20,21]. The maximum-likelihood (ML) core-genome phylogenetic tree was constructed using FastTree with 1000 bootstraps under the generalized time-reversible model and was then visualized using iTOL v6 (https://itol.embl.de/) [22].

The *A. baumannii* sequence type (ST) was assigned using both the Pasteur scheme [23] and the Oxford scheme [24] available at https://pubmlst.org/organisms/acinetobacter-baumannii. The capsular polysaccharide (CPS) [K locus (KL)] and lipooligosaccharide outer core locus (OCL) of the isolates were predicted using Kaptive v2.0.1 against the *A. baumannii* database (https://kaptive-web.erc.monash.edu/) [25].

The presence of antimicrobial resistance (AMR) genes in the assembled Terengganu *A. baumannii* genomes was predicted through the ResFinder 4.4.2 webserver (at http://genepi.food.dtu.dk/resfinder) where the input sequence was set at a 90% identity with a minimum length of 60% and the resistance gene identifier (RGI) from the Comprehensive Antibiotic Resistance Database (CARD) (https://card.mcmaster.ca/). The prediction of resistome from RGI (RGI 6.0.2 and CARD 3.2.7) was determined based on homology and SNP models. The criteria ‘perfect and strict hits only’ were chosen for the prediction. The virulence factor genes present in the sequenced *A. baumannii* isolates were predicted using the VFanalyzer against the *Acinetobacter* genus in the Virulence Factor Database (VFDB) updated in 2023 (http://www.mgc.ac.cn/cgi-bin/VFs/v5/main.cgi) [26]. The prediction of type VI secretion system (T6SS) core locus and four identified T6SS effectors in *A. baumannii* was determined by blast search against reference sequences (Data S1, available in the online version of this article) with 80% identity and 80% sequence coverage threshold used to select positive matches [27]. The plasmid *rep* genes were identified and characterized using blast analysis against the Acinetobacter Plasmid Typing database (https://github.com/MehradHamidian/AcinetobacterPlasmidTyping) [28]. CRISPR*-*Cas systems were detected using the CRISPRCasFinder (https://crisprcas.i2bc.paris-saclay.fr/) whereby only isolates with the highest level 4 CRISPRs were identified as CRISPR-Cas-positive isolates while those with the remaining lower level (i.e. levels 1–3) were considered as false CRISPRs [29].

The assembled draft genomes have been deposited in the NCBI database under the BioProject PRJNA573295.

### Concordance between phenotypic and genotypic resistance profiles

The concordance between the observed antimicrobial susceptibility phenotype and predicted genotype was assessed using Cohen’s kappa coefficient. The concordance was expressed by the kappa coefficient value and was interpreted as follows: values ≤0 indicated no agreement, 0.01–0.20 as slight, 0.21–0.40 as fair, 0.41–0.60 as moderate, 0.60–0.80 as substantial and 0.81–1.00 as almost perfect agreement [30]. The sensitivity and specificity for genotypic resistance prediction were calculated according to Katiyar *et al.* [31]. For analysis purposes, the susceptible and intermediate phenotypes were considered to be susceptible [32], and three antibiotic combinations, namely, piperacillin/tazobactam, ampicillin/sulbactam and trimethoprim/sulfamethoxazole, were dropped as they were used in combination.

## Results

### Phenotypic antimicrobial susceptibility

**Fig. 1.**
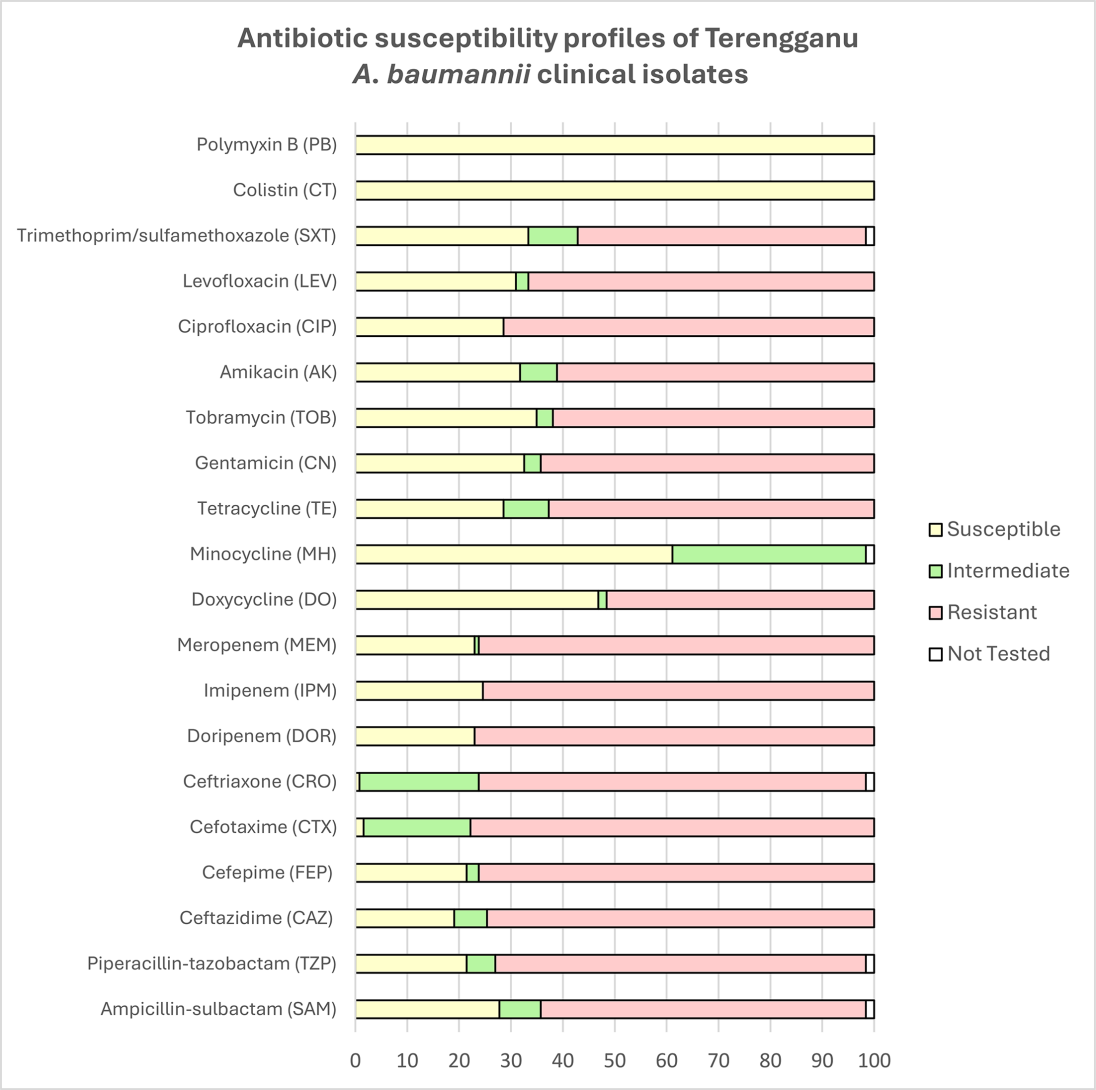
Antibiotic resistance profiles of the 126 *A*. *baumannii* clinical isolates from HSNZ, Terengganu, from 2011 to 2020.

The antimicrobial susceptibility profiles of the 126 Terengganu *A. baumannii* clinical isolates against 20 antibiotics belonging to 8 antimicrobial classes are shown in Fig. 1. The highest incidence of resistance was observed for cefotaxime (*n*=98; 77.8%) followed by doripenem (*n*=97; 77.0%), cefepime (*n*=96; 76.2%), meropenem (*n*=96; 76.2%), imipenem (*n*=95; 75.4%), ceftazidime (*n*=94; 74.6%), ceftriaxone (*n*=94; 74.6%), ciprofloxacin (*n*=90; 71.4%), piperacillin/tazobactam (*n*=90; 71.4%), levofloxacin (*n*=84; 66.7%), gentamicin (*n*=81; 64.3%), ampicillin/sulbactam (*n*=79; 62.7%), tetracycline (*n*=79; 62.7%), tobramycin (*n*=78; 61.9%), amikacin (*n*=77; 61.1%), trimethoprim/sulfamethoxazole (*n*=70; 55.6%) and doxycycline (*n*=65; 51.6%). All isolates were susceptible to colistin and polymyxin B. Ninety-seven (77.0%) of the isolates were categorized as MDR strains where they exhibited resistance towards three or more classes of antibiotics [16].

### Whole-genome sequencing analysis

#### Multilocus sequence typing

**Fig. 2.**
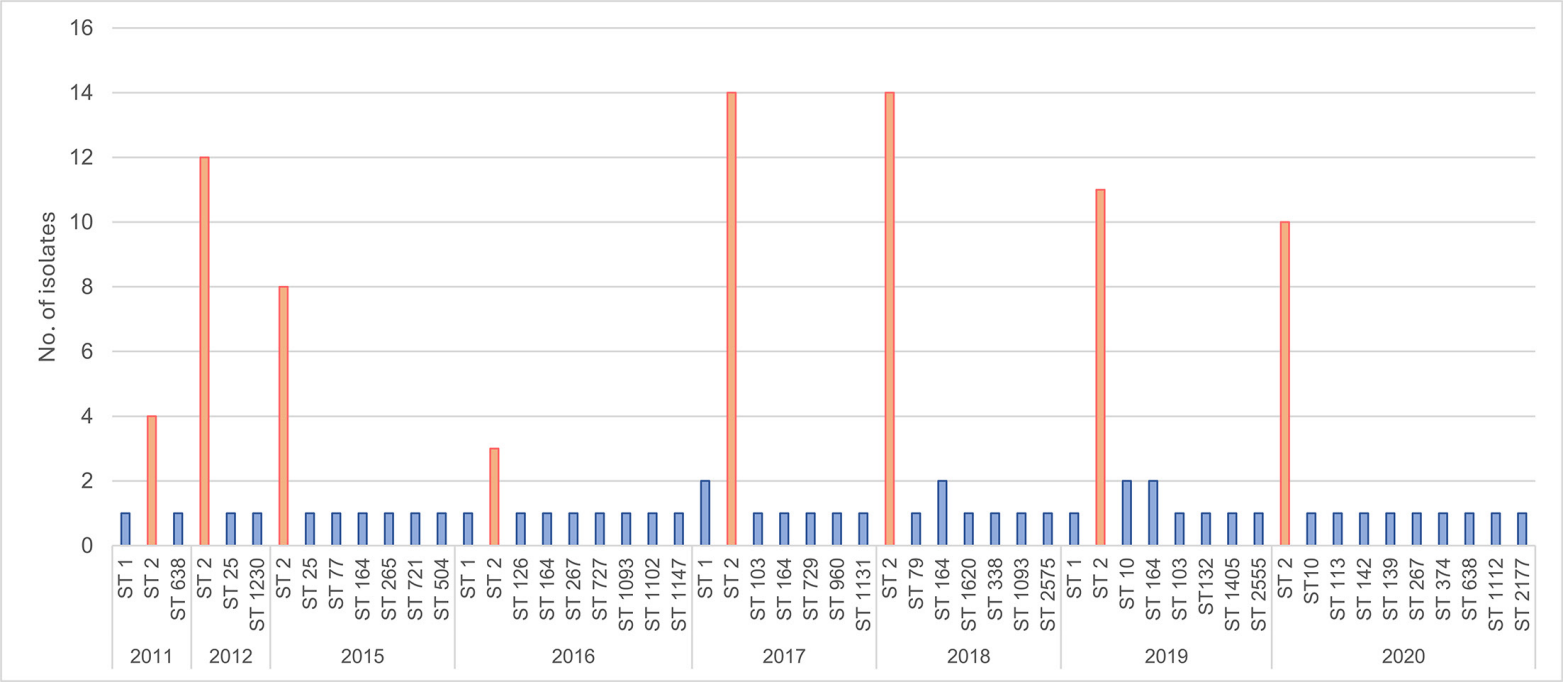
The distribution of Pasteur MLST by year of isolation for the 126 *A*. *baumannii* clinical isolates from HSNZ, Terengganu. The predominance of ST2_Pasteur_ (depicted in orange) in each year was evident.

The multilocus sequence typing (MLST) classified the Terengganu *A. baumannii* clinical isolates under 34 and 35 known Pasteur and Oxford STs, respectively (Data S1). Based on the Pasteur MLST scheme, ST2_Pasteur_ which belonged to the Global Clone 2 (GC2) lineage was identified as the dominant ST, with 60.3% (*n*=76/126) of the HSNZ *A. baumannii* clinical isolates belonging to this ST. The predominance of ST2_Pasteur_ among the *A. baumannii* isolates from HSNZ is demonstrated in Fig. 2, which showed that majority of the *A. baumannii* isolates obtained every year from the hospital were of this ST. Other STs detected were of not such high numbers, with ST164_Pasteur_ found at 5.6% (*n*=7/126) and ST1_Pasteur_ at 4.0% (*n*=5/126). This was also reflected in the minimum spanning tree of the Pasteur STs that were identified in this study (Fig. S1). ST164_Pasteur_ was recently grouped under the new International Clone (IC)/Global Clone 11 (GC11) [33], while ST1_Pasteur_ is a well-established GC1 lineage [34].

There were several issues associated with the Oxford scheme-based MLST, including recombination and the paralogy of the *gdhB* locus [35,37], leading to the identification of two Oxford STs in certain cases. Gaiarsa *et al*. [36] reported that the presence of two copies of the *gdhB* gene (designated *gdhB* and *gdhB2*) led to the incorrect establishment of new Oxford STs that do not actually exist, and the incorrect *gdhB2* alleles that were identified were 182 and 189. The authors recommended that these *gdhB2*-based alleles be removed from the database with the genomes re-analysed to exclude the paralogue *gdhB2* to determine the correct *gdhB* allele. We have done so with our isolates, and those with two Oxford STs either had the *gdhB2* allele 189 (Oxford ST1806, ST1809, ST1816, ST2149 and ST2805) or allele 182 (Oxford ST1604 and ST1840) and were removed from our analysis. We opted to include the Oxford STs despite the issues with this scheme as it was reported to be more discriminatory than the Pasteur scheme [36,37], and this was evident in our analysis of the ST2_Pasteur_ isolates in the upcoming section. The three most dominant Oxford STs in our isolates were ST195_Oxford_ (27.8%; *n*=35), ST208_Oxford_ (13.5%; *n*=17) and ST684_Oxford_ (7.1%; *n*=9).

Two isolates were assigned into new Pasteur STs, i.e. ST2555_Pasteur_ (AC1934) and ST2575_Pasteur_ (AC1839), and the corresponding Oxford STs, ST3349_Oxford_ (AC1934) and ST3348_Oxford_ (AC1839). Seven other isolates with existing Pasteur STs were assigned with novel Oxford STs (Data S1), whereas one ST2_Pasteur_ isolate, AC1601, could not be assigned with an Oxford ST due to low coverage and alignment length of two of the Oxford loci, i.e. *gpi* and *gyrB* (coverage of 60.9836% or 186/305 nt and 87.7462% or 401/457 nt, respectively).

#### Phylogenetic analysis

**Fig. 3.**
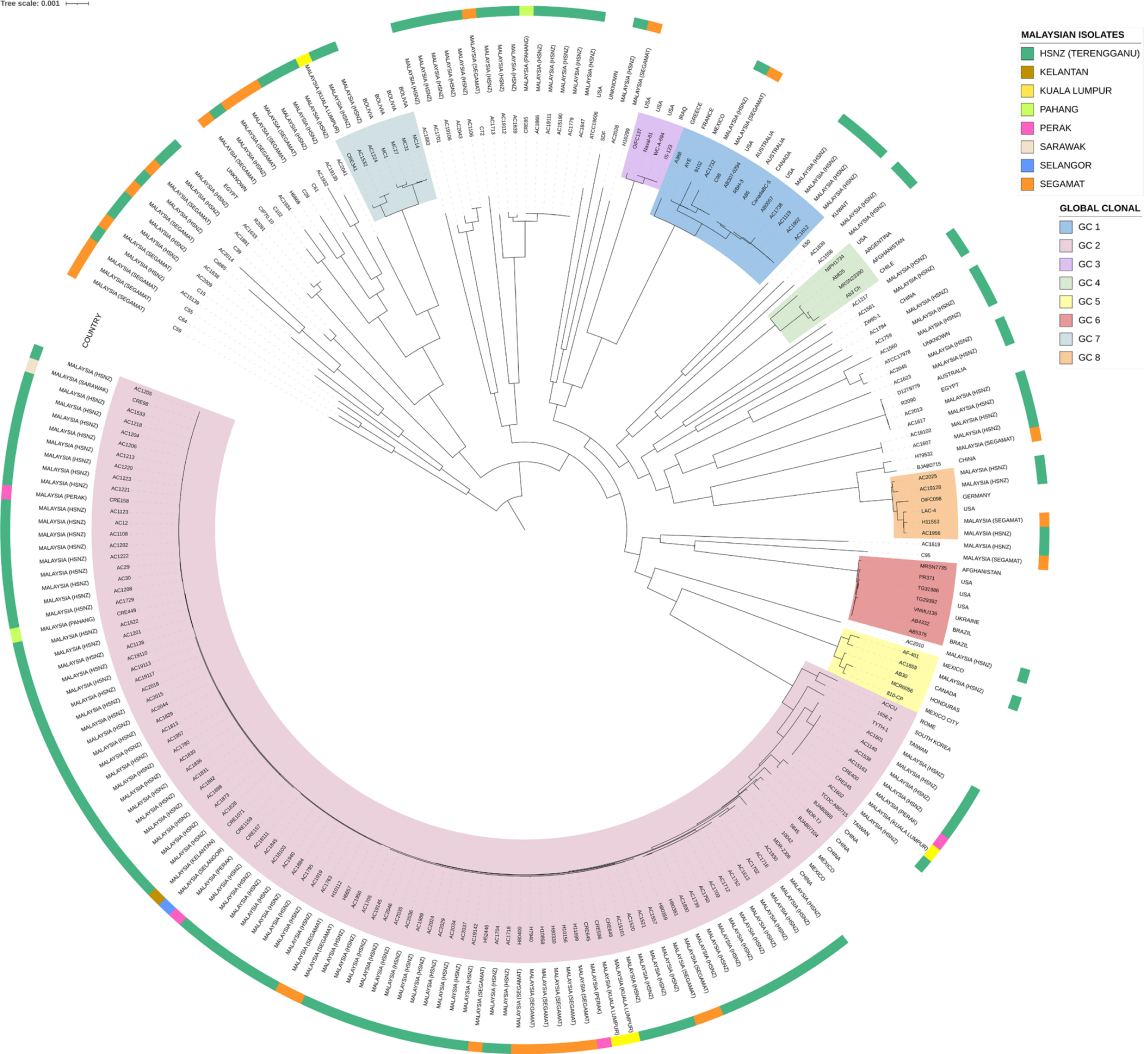
Midpoint-rooted ML core-genome phylogenetic tree of the Terengganu *A. baumannii* isolates together with other Malaysian *A. baumannii* genomes (as reported by Rao *et al.* [85] and Muzahid *et al.* [84] and indicated in the outermost ring of the tree as the towns and states within Malaysia) and several *A. baumannii* reference genomes representing known major GC types GC1 to GC8 as listed in Data S1.

A core-genome ML phylogenetic tree was constructed for the Terengganu *A. baumannii* isolates in comparison with other Malaysian *A. baumannii* genomes in the database along with 96 other *A. baumannii* reference genomes (Data S1) that represent the known major Global Clone (GC)/IC types ranging from GC1 to GC8 (Fig. 3). The phylogenetic tree clearly showed the prevalence of the GC2 lineage, especially ST2_Pasteur_, in HSNZ and in the other Malaysian *A. baumannii* genomes in the database. Nevertheless, other lineages were also identified in the HSNZ isolates, including GC1, GC5, GC7 and GC8, which were clustered in several clades in the phylogenetic tree (Fig. 3). There were also a few isolates that were not categorized into any one of the GC lineages. The ST2_Pasteur_ isolates that were collected from 2011 to 2020 were clustered closely on the same branch of the phylogenetic tree. To investigate these ST2_Pasteur_ isolates further, a separate phylogenetic tree and a minimum spanning tree based on their Oxford STs were constructed based on just the 76 ST2_Pasteur_ isolates along with 3 ST2_Pasteur_ genomes from the same hospital isolated in 2011 but that were sequenced and presented previously (i.e. AC12, AC29 and AC30) [38,39] (Figs S2 and S3). The average nucleotide identity (ANI) between these ST2_Pasteur_ isolates ranged from 99.76 to 100%, indicating their very close similarities. The ST2_Pasteur_ isolates could be further subdivided into the different Oxford STs with the largest group being ST195_Oxford_ (*n*=35+3 isolates from 2011 sequenced previously=38), followed by ST208_Oxford_ (*n*=17), ST684_Oxford_ (*n*=9), ST574_Oxford_ (*n*=7), ST938_Oxford_ (*n*=4) and ST547_Oxford_ (*n*=3). Nevertheless, one ST2 isolate, AC1601, had an unknown Oxford ST, as has been stated earlier.

#### AMR genes

**Fig. 4.**
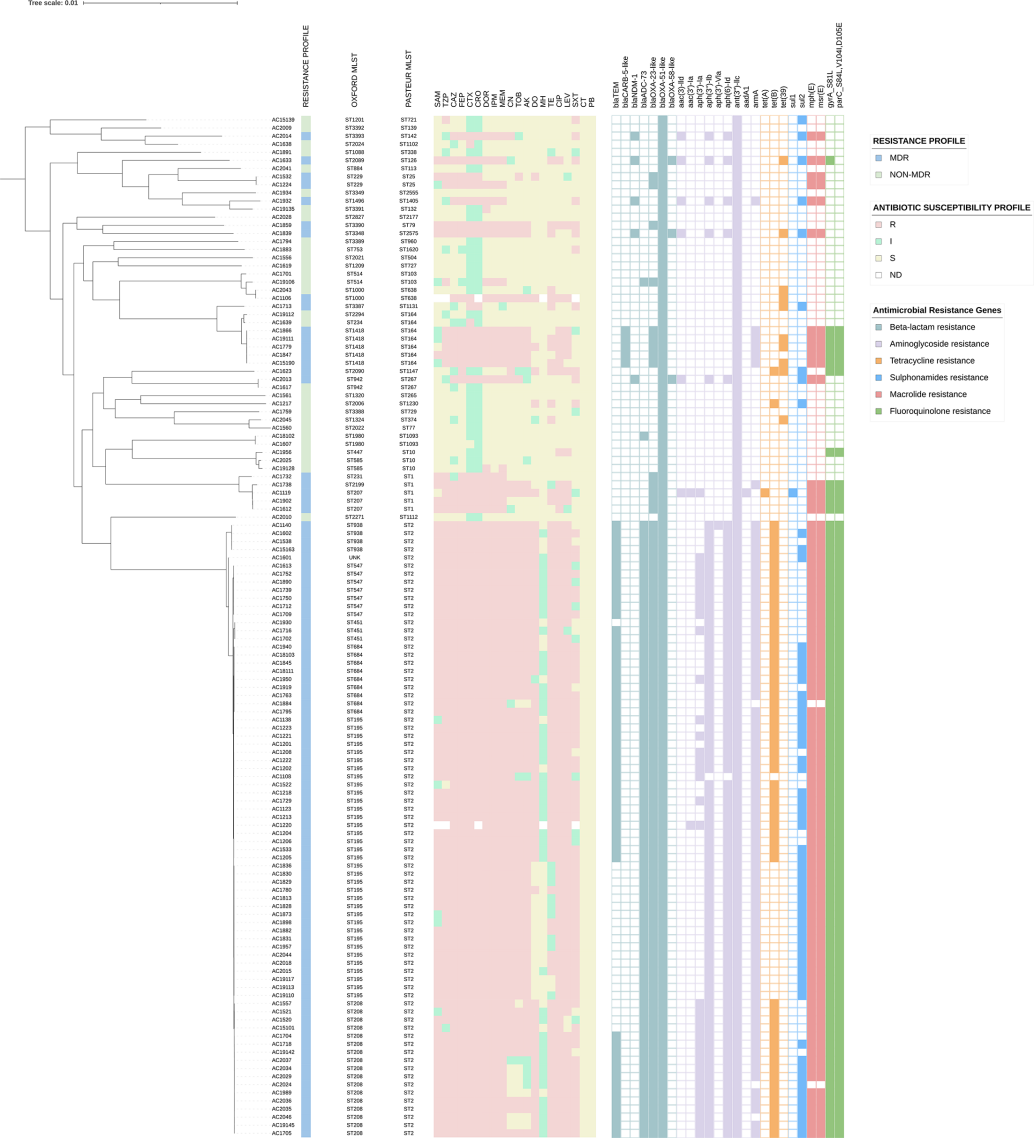
The phenotypic AMR profiles and corresponding carriage of AMR genes of the 126 Terengganu *A. baumannii* isolates plotted along with the ML core-genome phylogenetic tree of the isolates. Each isolate was also annotated with their corresponding Pasteur and Oxford MLST. The antibiotics are labelled as follows: ampicillin/sulbactam (SAM), piperacillin/tazobactam (TZP), ceftazidime (CAZ), cefepime (FEP), cefotaxime (CTX), ceftriaxone (CRO), doripenem (DOR), imipenem (IPM), meropenem (MEM), gentamicin (CN), tobramycin (TOB), amikacin (AK), doxycycline (DO), minocycline (MH), tetracycline (TE), ciprofloxacin (CIP), levofloxacin (LEV) and trimethoprim/sulfamethoxazole (SXT), polymyxin B (PB) and colistin (CT). The assigned AMR phenotypes are colour coded as light red for resistance, green for intermediate resistance and yellow for susceptible. The genotypic AMRs are sorted by antibiotic classes to which they confer resistance, and the colourless squares represent the absence of AMR genes. UNK indicates unknown.

Various AMR genes associated with resistance against several antimicrobial classes were detected in the Terengganu *A. baumannii* clinical isolates (Fig. 4). Genes encoding four Ambler classes of *β*-lactamases were identified across the 126 *A*. *baumannii* isolates with all isolates harbouring more than one oxacillinase genes. The class A extended-spectrum *β*-lactamase (ESBL) gene, *bla*_TEM_, was found in 54/126 (42.9%) isolates, all of which belonged to ST2_Pasteur_ and several Oxford STs (ST195, ST208, ST451, ST547, ST684 and ST938), while the *bla*_CARB-5-like_ genes were found in 5/126 (4.0%) isolates, all of which belonged to ST164_Pasteur_/ST1418_Oxford_ (Fig. 4). The class B metallo*-β*-lactamase (MBL), *bla*_NDM-1_, was found in 5/126 (4.0%) isolates, each of which belonged to different STs. The class C *β*-lactamase gene, *bla*_ADC_, which is mostly intrinsic and primarily responsible for cephalosporin resistance in * A. baumannii* [40] was found in the majority of the Terengganu *A. baumannii* isolates (*n=*119/126; or 94.4%). The most frequent Acinetobacter-derived cephalosporinase (ADC) variant identified in the isolates was ADC-73 (*n*=76) that differed in two aas to ADC-25 (i.e. G247S and N341T), which was reportedly found in members of the *A. baumannii* group and *Acinetobacter calcoaceticus* [41]. Two groups of acquired class D *β*-lactamases were identified in the Terengganu *A. baumannii* isolates, namely, *bla*_OXA-23-like_ (*n*=90/126; 71.4%) and *bla*_OXA-58-like_ (*n*=3/126; 2.4%). The three isolates that harboured the *bla*_OXA-58-like_ gene were of different STs and also co-harboured the *bla*_NDM-1_-encoded MBL (Fig. 4) . The intrinsic class D *β*-lactamase gene, *bla*_OXA-51-like_, was found in all the Terengganu *A. baumannii* isolates, and majority of them were identified as *bla*_OXA-66_ (*n*=76/126; 60.3%), all of which were ST2_Pasteur_. Five *A. baumannii* isolates harboured the *bla*_OXA-69_-encoded OXA-51-like variant and all five were ST1_Pasteur_, whereas three isolates that contained the *bla*_OXA-68_ variant were ST10_Pasteur_. The presence of class D *β*-lactamases was proposed to be characteristic of the *Acinetobacter* genus [41,42], and our data did not indicate otherwise. Interestingly, in all 126 * A*. *baumannii* isolates, IS*Aba1* (or any other insertion sequence element) was absent upstream of the *bla*_OXA-51-like_ gene, particularly in carbapenem-resistant isolates. This indicates that the overexpression of the *bla*_OXA-51-like_ gene due to the outward-directing promoter in IS*Aba1* and related elements was not a likely mechanism of carbapenem resistance in the Terengganu isolates, in support of the conclusions derived by Nigro and Hall [43].

Nine different aminoglycoside resistance genes were identified with the intrinsic aminoglycoside nucleotidyltransferase family gene, *ant(3″)-IIc*, detected in all the Terengganu *A. baumannii* clinical isolates. Eighty isolates (63.5%) also harboured the aminoglycoside phosphotransferase genes, *aph(3″)-Ib* and *aph(6)-Id*, while the *aph(3′)-Ia* gene was found in 36/126 (28.6%) and the *aph(3′)-VIa* gene was identified in just 1 isolate (0.8%), AC1140, which belonged to ST2_Pasteur_/ST938_Oxford_. The aminoglycoside acetyltransferase genes, *aac(3′)-Ia* and *aac(3)-IId*, were only found in 2/126 (1.6%) and 5/126 (4.0%) isolates, respectively. Interestingly, all five isolates that harboured the *aac(3)-IId* gene also co-harboured the *bla*_NDM-1_ MBL gene and were of different STs (Fig. 4). The two isolates that encoded the *aac(3′)-Ia* gene were similarly of different STs (AC1119 – ST1_Pasteur_/ST2199_Oxford_ and AC1220 – ST2_Pasteur_/ST195_Oxford_). AC1119 is also the only isolate in our collection that harboured the aminoglycoside adenylyltransferase gene, *aadA*. The 16S rRNA methylase gene, *armA*, was identified in 74/126 (58.7%) isolates.

Two resistance genes that confer resistance to sulphonamides were identified in the Terengganu *A. baumannii* isolates with the *sul2* gene found in 61/126 (48.4%) isolates, whereas only a single isolate, AC1119, harboured the *sul1* gene (Fig. 4). The macrolide resistance genes, *msrE* and *mphE*, were observed to co-exist in 90/126 (71.4%) isolates. Three genes encoding resistance to tetracyclines were identified in the Terengganu isolates, with *tet(B*) being the most prevalent at 60/126 (47.6%) isolates followed by *tet(39)* (*n*=10/126; 7.9%) and *tet(A*), which was found in only a single isolate, AC1119 (0.8%). Resistance to fluoroquinolones in the *A. baumannii* isolates was promoted by aa mutations in the QRDRs of the target sites in DNA gyrase and DNA topoisomerase IV. The S81L mutation in the *gyrA-*encoded DNA gyrase was found in 88/126 (69.9%) of the isolates, and in all these isolates, the S84L, V104I and D105E mutations were also identified in the *parC-*encoded DNA topoisomerase IV except for AC1633, which had only the V104I and D105E mutations. These two mutations in ParC were also identified in the remaining *A. baumannii* isolates (Data S1).

#### Concordance between phenotypic and genotypic resistance profiles

The concordance between the predicted genotype and the resistance phenotype obtained by the AST was determined using Cohen’s kappa coefficient of agreement for 16 antibiotics covering 6 antibiotic classes (Table 1). The intermediate phenotypes were counted as susceptible in this analysis [32]. The concordance was expressed by the kappa (*κ*) coefficient value where almost perfect agreement between genotype and phenotype (*κ* value=0.81–1.00) was observed for nine of the tested antibiotics, i.e. doripenem, imipenem, meropenem, doxycycline, tetracycline, tobramycin, amikacin, ciprofloxacin and levofloxacin. Substantial agreement (*κ* value=0.6–0.80) was observed for ceftazidime, cefepime, cefotaxime, ceftriaxone and gentamicin, whereas no agreement was found for minocycline due to the absence of observed resistance phenotypes among the HSNZ *A. baumannii* isolates.

**Table 1.** The concordance between the predicted genotype and the resistance phenotype of the HSNZ *A. baumannii* isolates

Antimicrobial class and drugs		Phenotype: resistance		Phenotype: susceptible		Cohen’s kappa (*κ*)(95 % CI)	Agreement	Resistance gene(s)
		Genotype:resistant	Genotype:susceptible	Genotype:resistant	Genotype:susceptible			
Cephalosporins	CAZ	81	13	0	32	0.760 (0.640 to 0.880)	Substantial	*bla*_ADC-73_, *bla*_TEM_, *bla*_NDM_
	FEP	81	15	0	30	0.720 (0.592 to 0.848)	Substantial	
	CTX	81	17	0	28	0.679 (0.545 to 0.814)	Substantial	
	CRO	80	14	0	30	0.734 (0.608 to 0.861)	Substantial	
Carbapenems	DOR	94	3	1	28	0.913 (0.828 to 0.997)	Almost perfect	*bla*_NDM_, *bla*_OXA-23_, *bla*_OXA-58_, *bla*_CARB_
	IPM	94	1	1	30	0.957 (0.898 to 1.000)	Almost perfect	
	MEM	94	2	1	29	0.935 (0.863 to 1.000)	Almost perfect	
Tetracyclines	DO	62	3	8	53	0.825 (0.726 to 0.923)	Almost perfect	*tet(A*), *tet(B*), *tet(39)*
	TE	70	9	0	47	0.853 (0.761 to 0.945)	Almost perfect	
	MH	0	0	65	59	0	No agreement	*tet(B*)
Aminoglycosides	CN	78	3	9	36	0.786 (0.672 to 0.900)	Substantial	*aac(3′)-Ia*, *aac(3)-lld*, *aph(3′)-la*, *aph(3′)-Vla*, *armA*
	TOB	72	6	2	46	0.868 (0.779 to 0.956)	Almost perfect	*armA*
	AK	73	4	1	48	0.917 (0.847 to 0.988)	Almost perfect	*aph(3′)-Vla*, *armA*
Fluoroquinolones	CIP	87	3	0	36	0.943 (0.880 to 1.000)	Almost perfect	*gyrA* S81L and *parC* S84L, V104I, D105E
	LEV	82	2	5	37	0.873 (0.781 to 0.964)	Almost perfect	

AKamikacinCAZceftazidimeCIPciprofloxacinCNgentamicinCROceftriaxoneCTXcefotaximeDOdoxycyclineDORdoripenemFEPcefepimeIMPimipenemLEVlevofloxacinMEMmeropenemMHminocyclineTEtetracyclineTOBtobramycin

#### Plasmid identification

**Fig. 5.**
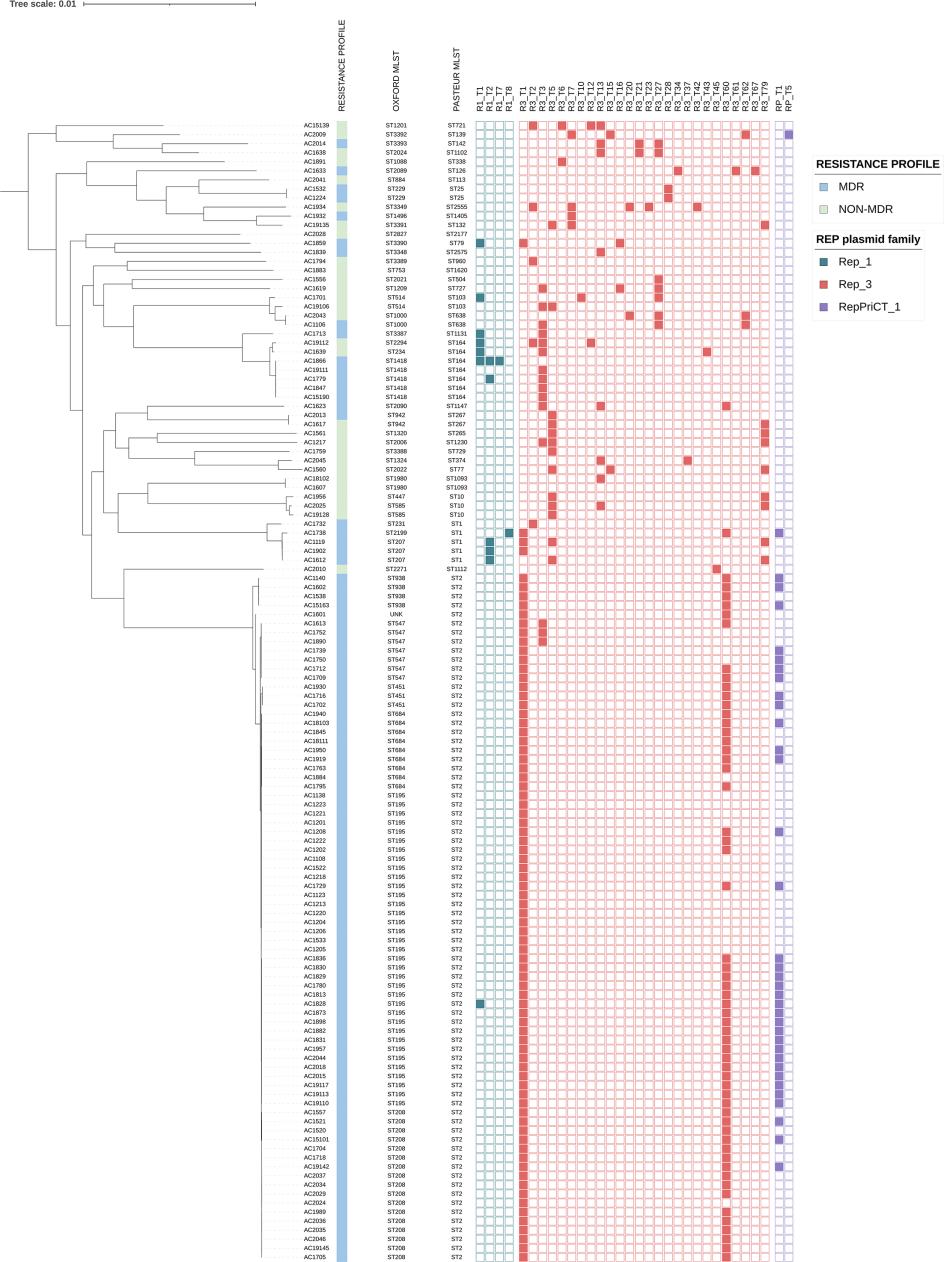
Carriage of potential plasmids in the 126 sequenced Terengganu *A. baumannii* isolates. The genome sequences were screened for the presence of the *Acinetobacter* plasmid-encoded replicase genes, which were classified either as the Rep_1 (blue squares), Rep_3 (red squares) or PriCT_1 (purple squares) families and further subtyped as detailed in [28] and [46]. Each isolate was also annotated with their corresponding Pasteur and Oxford STs, and whether they are categorized as MDR isolates.

Plasmid-encoded *rep* genes were identified in 122/126 (96.8%) of the Terengganu *A. baumannii* isolates with none detected in 4 non-MDR isolates, i.e. AC1607, AC1883, AC2028 and AC2041 (Fig. 5, Data S1). Most of the isolates (*n*=90/126; 71.4%) carried 2 to a maximum of 5 types of plasmid *rep* genes, while the remaining 32/126 (25.4%) isolates carried a single plasmid *rep* gene. The Acinetobacter Plasmid Typing scheme established by Lam *et al*. in 2023 [28] and expanded by the same group in 2024 [44] categorizes *Acinetobacter* plasmids into three main families, i.e. Rep_1, Rep_3 and RepPriCT_1, based on their Pfam domains of the *rep-*encoded replication initiation protein. All three families were detected in the Terengganu *A. baumannii* isolates with the majority being the Rep_3 family, which was identified in nearly all (*n*=121/126; 96%) of the isolates (Fig. 5). Of the Rep_3 family plasmids, the Rep_3-T1 (*n*=80/126; 63.5%) and the Rep_3-T60 (*n*=58/126; 46.0%) subgroups were the most predominant. A majority of the ST2_Pasteur_ isolates (*n*=56/76; 73.7%) were found to co-harbour the two *rep* gene subgroups, as had been previously reported in ST2_Pasteur_ isolates from Thailand [45]. All the Terengganu *A. baumannii* isolates that were identified with the Rep_3-T1 subgroup harboured an identical 8731 bp plasmid that was designated pAC12 and that was found in three ST2_Pasteur_ isolates from the same hospital in 2011 [38]. This plasmid was identical to pAB0057 and pA1-1 that were classified under the previous *Acinetobacter* plasmid grouping GR2 [46,47], and these plasmids were found in numerous isolates belonging to GC1 and GC2 lineages [28]. pA1-1 was identified in the oldest GC1 isolate, *A. baumannii* A1 from the UK in 1982 [48], inferring a long association of the plasmid with these clones [28]. The Rep_3 family was also found in three other GC lineages in the Terengganu *A. baumannii* isolates, i.e. GC5, GC7 and GC8 (Fig. 5), which was in agreement with Lam and Hamidian [44] who reported the presence of the Rep_3 family plasmids carrying AMR genes in all major GCs of *A. baumannii*.

#### Virulence factors

**Fig. 6.**
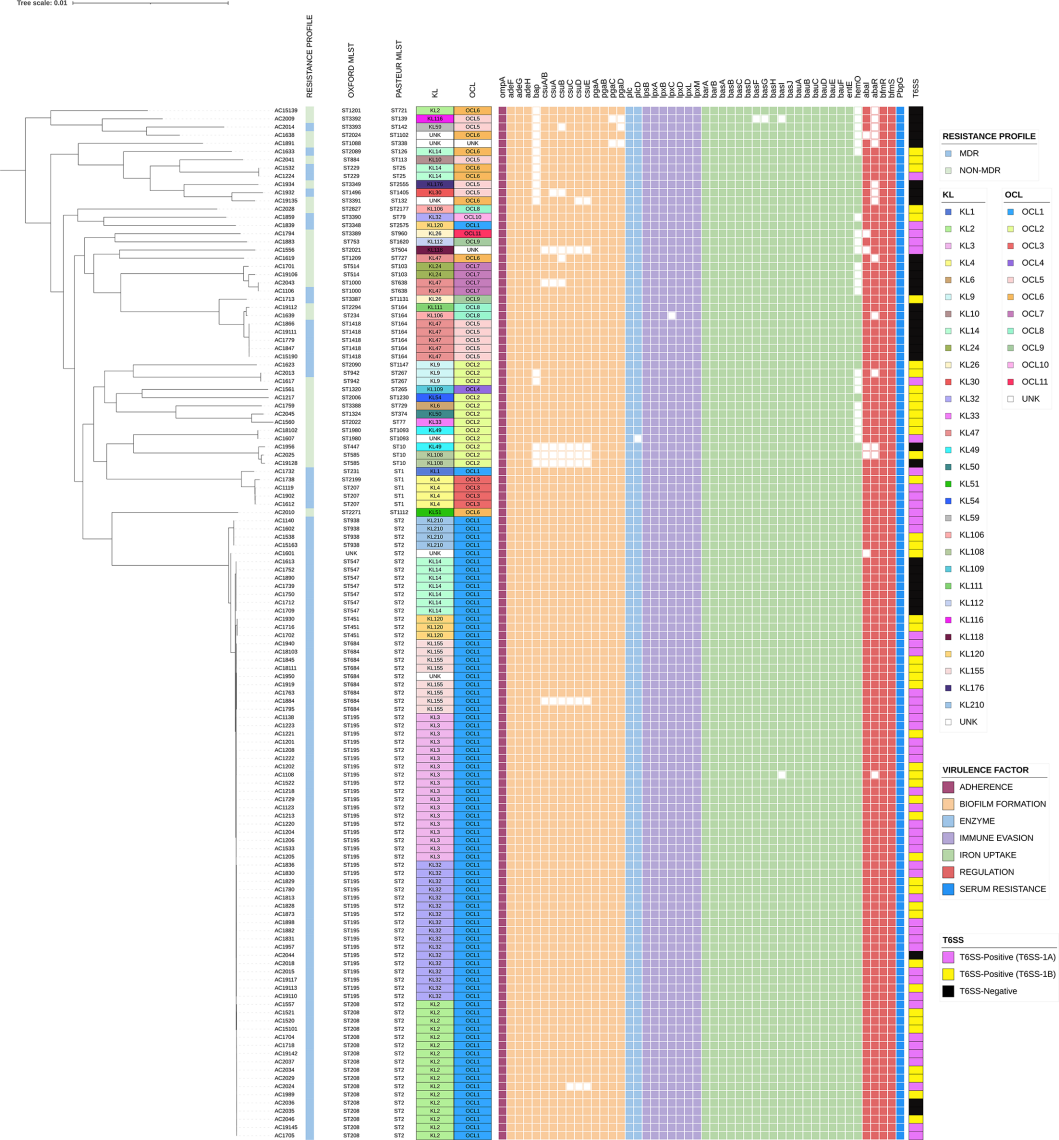
The ML core-genome phylogenetic tree of 126 Terengganu *A. baumannii* isolates was constructed using FastTree with 1000 bootstraps under the generalized time-reversible model and was then visualized using iTOL (https://itol.embl.de/). The tree was annotated with resistance profile, Oxford MLST, Pasteur MLST, the CPS chromosomal KL and OCL biosynthesis types and the virulence factor genes. The virulence genes are sorted by their virulence factors, and the colourless squares represent the absence of virulence genes. UNK indicates unknown.

All 126 sequenced *A. baumannii* isolates were examined for genes encoding virulence factors from the VFDB, and the results are depicted in Fig. 6. Similar virulence genes were present in almost all isolates, which suggests their pathogenic nature.

The outer membrane protein A gene, *ompA*, was identified in all the *A. baumannii* clinical isolates. The OmpA protein is located on the cell surface and acts as one of the major *A. baumannii* virulence factors by mediating the adhesion and invasion of the pathogen to epithelial cells [49]. The ability to form biofilms is considered as another important determinant of virulence in * A. baumannii* as biofilms represent a mechanism of resistance against antimicrobial treatments and host defences [50]. * A. baumannii* demonstrated strong biofilm formation capability not only on biotic surfaces (skin, wound and soft tissue) but also on abiotic surfaces in hospitals (artificial heart valves, catheters, intubation tubes and cleaning instruments) [51,53]. Here, a total of 14 genes associated with biofilm formation in VFDB were found in the Terengganu *A. baumannii* isolates including the *adeFGH-*encoded efflux pump, the *bap-*encoded biofilm-associated protein, *csuA/B-csuABCDE*-encoded Csu pili chaperone–usher assembly system and the *pgaABCD* genes that encode the synthesis of the intercellular adhesin poly-β-(1,6)-*N*-acetyl glucosamine. Although present in all the Terengganu ST2_Pasteur_ isolates, the *bap* gene was not detected in 17 of the *A. baumannii* Terengganu isolates, including strains that belonged to ST25_Pasteur_, ST267_Pasteur_ and ST10_Pasteur_, among others (Fig. 6). Besides the absence of the *bap* gene, genes for the Csu pili were also not found in the three ST10_Pasteur_ isolates (i.e. AC1956, AC2025 and AC19128). The solitary ST504_Pasteur_ isolate, AC1556, and one of the ST2_Pasteur_ isolates, AC1884, were also missing the entire Csu pili genes but retained the *bap* gene (Fig. 6).

*A. baumannii* produces the siderophore acinetobactin to gain iron from iron-limited host habitats [54]. The siderophore efflux system (*barAB*), the genes involved in acinetobactin uptake (*bauABCDEF*) and the iron uptake-related gene *entE* were detected in all 126 Terengganu *A. baumannii* isolates, while the complete 9 genes representing the acinetobactin biosynthesis cluster (*basABCDFGHIJ*) were identified in 124/126 (98.41%) of the isolates (Fig. 6). The putative haem oxygenase encoded by the *hemO* gene that was responsible for haem uptake and utilization was identified in 104/126 (82.54%) of the isolates. Previously, the *hemO* gene cluster has been identified in ~ 60% of *A. baumannii* clinical strains [55]. Serum resistance is another important virulence determinant in *A. baumannii* that enables this pathogen to survive in the human bloodstream leading to severe infections [56]. Here, the *pbpG* gene that encodes a penicillin-binding protein responsible for serum resistance in *A. baumannii* was identified in all 126 Terengganu isolates.

The T6SS is another virulence factor utilized by *A. baumannii* to release toxic effector proteins into neighbouring bacterial or eukaryotic cells, thus allowing the bacteria to spread, invade and resist the host immune responses [57,59]. Consequently, T6SS may offer a competitive advantage to *A. baumannii* in multispecies environments [60]. In the *A. baumannii* genome, a minimum of 13 highly conserved proteins encoded in a single locus formed the core T6SS component, which includes genes that encode for a baseplate (*tssAEFGK*), a membrane-linked stabilizing structure (*tssMJL*), a contractile sheath (*tssBC*), a cytoplasmic sheath recycling protein (*tssH*) and an injectable inner tube (*hcp*, *vgrG* and PAAR) [61,63]. The complete *A. baumannii* T6SS core locus (*tssA*, *tssB*, *tssC*, *tssE*, *tssF*, *tssG*, *tssH/clpV*, *tssK*, *tssL*, *tssM*, *tagX*, *hcp*, *vgrG* and PAAR) was identified in 94/126 (74.60%) isolates (Fig. 6; Data S1). Previous bioinformatic analyses have predicted the presence of two transcriptional blocks in the T6SS loci, and the separation of this locus into two transcriptional units led to the existence of two different genetic arrangements designated as T6SS-1A and T6SS-1B among the *A. baumannii* T6SS gene clusters [64]. In this study, 49/94 (52.1%) of the T6SS-positive * A. baumannii* strains belonged to T6SS-1A, while the remaining 45/94 (47.9%) were T6SS-1B (Fig. 6).

In the Terengganu *A. baumannii* isolates, 15/126 (11.9%) of the isolates contain at least 1 of the recognized T6SS effector genes. These effectors elicit different degrees of response, usually lysis, of the target competing cells [64]. The four T6SS effectors identified in the *A. baumannii* isolates were Tse1 (a predicted lipase effector), Tse2 (a predicted nuclease effector), Tse3 (an effector of unknown function) and Tse4 (a bifunctional enzyme predicted to contain amidase/PGase activity) [65]. Two of the Terengganu *A. baumannii* isolates (AC2035 and AC2036) were identified as T6SS-negative strains although they have the complete T6SS genes due to the separation of the genes into two different loci. In *A. baumannii*, the genes encoding the core T6SS component proteins are located in a single conserved locus [57,66].

#### Chromosomal KL and lipooligosaccharide OCL CPS biosynthesis genes

In addition to the virulence factors identified by the VFDB, the cell surface or CPS exhibited by *A. baumannii* has also been classified as important virulence determinant and is responsible in inducing the pathogenicity of *A. baumannii* isolates [67,68]. Two loci with variable gene clusters responsible for the production of CPS have been used as epidemiological markers for * A. baumannii* and were identified using the Kaptive database [69]. The chromosomal KL, located between the *fkpA* and *lldP* genes, is responsible for the biosynthesis and export of CPS, while the group of genes in the OCL, located between the *aspS* and *ilvE* genes, encodes the lipooligosaccharide OCL of this pathogen [69,70].

High diversity of KL was observed for the 126 Terengganu *A. baumannii* isolates where 30 different KL variants were identified. The most prevalent KL types were KL2, KL3 and KL32, which were each identified in 18/126 (14.29%) isolates, followed by KL14 in 10/126 (7.94%) isolates; KL47 and KL155 in 8/126 (6.35%) isolates; and KL4, KL120 and KL210, each of which were found in 4/126 (3.17%) isolates. The other KL variants were found in three or fewer isolates. On the other hand, OCL was more conserved among the Terengganu *A. baumannii* isolates, with only 11 variants identified. The majority of the *A. baumannii* isolates belonged to OCL1 (*n*=78/126; 61.90%), followed by OCL2 (*n*=12/126; 9.52%), OCL5 (*n*=10/126; 7.94%), OCL6 (*n*=8/126; 6.35%) and OCL7 (*n*=4/126; 3.17%). The remaining seven OCL variants were found in three or fewer isolates. The distribution of KL and OCL types in the Terengganu *A. baumannii* is indicated in Fig. 6.

It is interesting to note that within the ST2_Pasteur_ Terengganu isolates, all of them belonged to OCL1, but their KL types corresponded to their Oxford STs (Figs 6, S2 and S3). KL2 was identified in 17 ST2_Pasteur_ isolates, and all 17 belonged to ST206_Oxford_. Similarly, eight isolates belonged to KL155, and all eight were ST654_Oxford_ (with one exception whereby the KL type was unknown for AC1950); seven isolates were KL14, and all seven were ST547_Oxford_; four isolates were KL210 and all four were ST938_Oxford_; and three isolates were KL120, and all three were ST451_Oxford_. The exception to this observation was for ST2_Pasteur_/ST195_Oxford_ where these isolates were either KL3 (*n*=18) or KL32 (*n*=17) (Figs 6, S2 and S3). Interestingly, the ST195_Oxford_-KL32 isolates only appeared from 2017 onwards, whereas the ST195_Oxford_-KL3 isolates were obtained mainly from 2011 to 2015 with only one isolate found in 2017. The other predominant subgroup of ST2_Pasteur_, ST208_Oxford_-KL2, was only found from 2015 onwards, with seven isolates from 2020 belonging to this subgroup, inferring possible nosocomial spread as five of these isolates were obtained from the same ward (i.e. ward 8) (Figs S2 and S3).

In our collection of 126 *A. baumannii* isolates, only 6 (4.8%) had 0 confidence matches against the KL database and 2 (1.6%) against the OCL database. This might be due to the presence of other genes in the locus that are unavailable in the current Kaptive database and therefore could be indicative of novel locus types [69].

#### The CRISPR-Cas system is present in all ST1_Pasteur_ isolates but absent in ST2_Pasteur_ isolates

The CRISPR*-*Cas system is one of the bacterial immune defence systems that helps in protecting the bacterial genome against the invasion of mobile genetic materials including phages [71]. Previously, CRISPR-Cas systems were categorized into 5 types and 16 subtypes [72] before a new CRISPR-Cas classification scheme was published in 2020, updating the classification into 2 classes, 6 types and 33 subtypes [73]. In general, the CRISPR-Cas type I-F system is identified by the presence of a unique fusion of two *cas* genes, *cas3/cas2*, which together with *cas1* form a complex in facilitating the integration of the spacer into the CRISPR locus [74,75]. In *A. baumannii* genomes, two CRISPR-Cas type I-F systems have been previously identified and were referred to as type I-Fa (in which the *csy1* gene is absent) and type I-Fb [29,75].

In this study, only 14/126 (or 11.1%) of the Terengganu *A. baumannii* isolates were identified as CRISPR-Cas-positive genomes. The characteristics of the CRISPR-Cas-positive isolates are summarized in Table 2. Majority of the CRISPR-Cas-positive Terengganu *A. baumannii* isolates (*n*=11) harboured a locus consisting of two CRISPR-associated genes (*cas1* and *cas3/cas2*), four Cas system-associated genes (*csy1*, *csy2*, *csy3* and *csy4*) and an array of spacers and were thus assigned as CRISPR-Cas type I-Fb. Another four isolates were identified with type I-Fa, and one isolate, AC1891 (ST338_Pasteur_), was found to harbour both types I-Fa and I-Fb (Table 2). The coexistence of both CRISPR-Cas types had been previously reported in 3.7% of *A. baumannii* genomes [76]. A closer look at one of the isolates identified with harbouring a type I-Fa, AC1759, showed the absence of the *cas3/cas2* gene, indicating the likelihood of an incomplete CRISPR-Cas system in the isolate (Table 2). The CRISPR-Cas-positive *A. baumannii* isolates belonged to nine Pasteur STs, including ST1_Pasteur_ (all five isolates of this ST, which were CRISPR-Cas type I-Fb) in agreement with reports that the GC1 lineage is a well-known CRISPR-Cas-positive lineage in *A. baumannii* [77,78]. None of the prevalent ST2_Pasteur_ isolates were found to harbour any CRISPR-Cas systems. There were two ST25_Pasteur_ isolates in our collection, and both were positive for CRISPR-Cas type I-Fb. In terms of phenotypic resistance, nine of the CRISPR-Cas-positive isolates, including the five ST1_Pasteur_ isolates and two ST25_Pasteur_ isolates, were categorized as MDR. AC1891, which harboured both types I-Fa and I-Fb, was non-MDR.

**Table 2.** List of CRISPR-Cas-positive Terengganu *A. baumannii* isolates identified in this study, their CRISPR-Cas type, along with their STs, and resistance profiles

No.	Isolate	MLST		Resistance profile	CRISPR-Cas type	Carriage of *cas* genes
		Pasteur	Oxford			
1	AC1119	ST1	ST207	MDR	Type I-Fb	*cas1-cas3/cas2-csy1-csy2-csy3-cas6*
2	AC1224	ST25	ST229	MDR	Type I-Fb	*cas1-cas3/cas2-csy1-csy2-csy3-cas6*
3	AC1532	ST25	ST229	MDR	Type I-Fb	*cas1-cas3/cas2-csy1-csy2-csy3-cas6*
4	AC1612	ST1	ST207	MDR	Type I-Fb	*cas1-cas3/cas2-csy1-csy2-csy3-cas6*
5	AC1633	ST126	ST2089	MDR	Type I-Fb	*cas1-cas3/cas2-csy1-csy2-csy3-cas6*
6	AC1732	ST1	ST231	MDR	Type I-Fb	*cas1-cas3/cas2-csy1-csy2-csy3-cas6*
7	AC1738	ST1	ST2199	MDR	Type I-Fb	*cas1-cas3/cas2-csy1-csy2-csy3-cas6*
8	AC1759	ST729	ST3388	Non-MDR	Type I-Fa (partial)	*cas1-cas6-csy3-csy2*
9	AC1794	ST960	ST3389	Non-MDR	Type I-Fa	*cas3/cas2-csy2-csy3-cas6-cas1*
10	AC1859	ST79	ST3390	MDR	Type I-Fa	*cas1-cas3/cas2-cas6-csy2-csy3*
11	AC1891	ST338	ST1088	Non-MDR	Type I-Fa	*cas1-cas3/cas2-cas6-csy2-csy3*
					Type I-Fb	*cas1-cas3/cas2-cas6-csy1-csy2-csy3*
12	AC1902	ST1	ST207	MDR	Type I-Fb	*cas1-cas3/cas2-csy1-csy2-csy3-cas6*
13	AC1932	ST1405	ST1496	MDR	Type I-Fb	*cas1-cas3/cas2-cas6-csy1-csy2-csy3*
14	AC2041	ST113	ST884	Non-MDR	Type I-Fb	*cas1-cas3/cas2-csy1-csy2-csy3-cas6*

## Discussion

Infections due to carbapenem-resistant *A. baumannii* have become a major healthcare concern since most carbapenem-resistant isolates are also resistant to multiple antimicrobials, thus severely limiting therapeutic options [79]. Characterizing and understanding the mechanisms of AMR and their evolution, particularly through genomics, in carbapenem-resistant *A. baumannii* and other MDR bacterial pathogens is an important first step in finding ways to mitigate their spread [80]. Whole genome sequencing (WGS) was thus used to investigate the genomic epidemiology of 126 *A. baumannii* clinical isolates obtained from HSNZ, the main tertiary hospital in Terengganu, Malaysia, over a 10-year period from 2011 to 2020.

We had previously reviewed the AMR profiles of clinical *Acinetobacter* spp. from Malaysia over a period of nearly three decades (1987–2016) [81]. From the very few reports that were available, the incidence of carbapenem resistance of *Acinetobacter* spp. in Malaysia in the 1980s was less than 5%, but this increased considerably to around 30–40% a decade later [81]. By the mid 2010s, the national incidence of carbapenem-resistant *A. baumannii* was around 50–60% although several studies from individual hospitals showed higher occurrences of carbapenem resistance [81]. Our previous studies of *A. baumannii* isolates from HSNZ showed that >70% of the isolates (*n*=54) were carbapenem resistant in 2011 (imipenem=74.1%; meropenem=77.8%) [82], whereas of the 128 isolates obtained in 2015, 68.8% (*n*=88) were carbapenem resistant [14]. These numbers were not that far off from the overall 74.6% (*n*=94/126) incidence of carbapenem resistance that were obtained for all 126 isolates obtained throughout 2011–2020 that were sequenced in this study. Nearly all the carbapenem-resistant isolates were also MDR in this study as well as in our earlier studies [81,82]. A recent 9-month (October 2019–July 2020) survey of ESKAPE pathogens from the University of Malaya Medical Centre in Kuala Lumpur, the capital city of Malaysia, alarmingly showed that all 54 *A. baumannii* isolates obtained were carbapenem resistant and 89% (*n*=48/54) of the isolates were MDR [83]. Of concern, 10 of the 54 isolates (or 19%) were colistin resistant, with colistin being one of the last-resort drugs for the treatment of MDR *A. baumannii* infections [83]. Muzahid *et al.* [84] also reported incidences of colistin (*n*=2/15) and polymyxin B resistance (*n*=4/15) among the *A. baumannii* isolates obtained from Hospital Segamat in the southern Malaysian state of Johor in 2020. No colistin-resistant isolate was found in this study although in an earlier study, we had reported a high 25% incidence of polymyxin resistance among 54 HSNZ * A. baumannii* isolates from 2011 [82].

The majority of the Terengganu *A. baumannii* were ST2_Pasteur_, which belonged to the GC2/IC2 lineage. This predominance was seen in our collection of isolates every year from 2011 to 2020 (Fig. 2). Two other papers that report on the genomes of * A. baumannii* Malaysian isolates also inferred this predominance although their reports were with smaller number of isolates. Of the 13 isolates that were collected from various states in Malaysia, Rao *et al.* [85] showed that 10 were of GC2 while Muzahid *et al.* [84] reported that in a collection of 15 hospitals plus 12 community isolates from the district of Segamat, located in the southern state of Johor, Malaysia, 11 of the 15 hospital isolates were GC2 while none of the community isolates were of that lineage. ST2_Pasteur_ is also the largest and most predominant ST in neighbouring Southeast Asian countries such as Thailand and Myanmar [40,86,89]. Hamidian and Nigro [34] reported the overwhelming predominance of ST2_Pasteur_ in their survey of carbapenem-resistant *A. baumannii* genomes that were available in the GenBank non-redundant and WGS databases as of April 2019 (whereby ST2_Pasteur_ comprised 59% of the 3609 genomes investigated, followed far behind by ST1_Pasteur_ of the GC1 lineage, which comprised 4.2%). The global distribution of carbapenem-resistant *A. baumannii* was thus heavily influenced by the spread of GC2 and, in particular, ST2_Pasteur_ isolates [34]. In this study, only five isolates were ST1_Pasteur_, and interestingly, Muzahid *et al.* [84] reported one ST1_Pasteur_ isolate from the community in Segamat, Malaysia. The ST2_Pasteur_ isolates from HSNZ were very closely related, with ANI values that ranged from 99.76 to 100%, and all belonged to OCL1. However, these ST2_Pasteur_ isolates could be further subdivided according to their Oxford STs and the KL types, with ST195_Oxford_-KL3 being the most predominant (*n*=21) followed by ST195_Oxford_-KL32 (*n*=17) and ST208_Oxford_-KL2 (*n*=17) (Figs S2 and S3), thus suggesting that their prevalence could not be due to the spread of a single epidemic strain. The predominance of ST195_Oxford_ was also reported by Rao *et al.* [85] with 5 of their 13 sequenced Malaysian *A. baumannii* isolates. In contrast, the ST195_Oxford_ clone was not found in any of the 15 sequenced hospital isolates from Segamat, Malaysia, but ST208_Oxford_ was predominant instead [84]. Nevertheless, any conclusions should be made with caution due to the small number of isolates from these two studies [84,85].

Interestingly, our *A. baumannii* collection contained seven ST164_Pasteur_ isolates, which were recently recognized as belonging to a new clonal lineage designated as GC11 [33]. Recent published data indicated that ST164_Pasteur_ has spread globally over the past decade but with low prevalence [90] although a longitudinal study in an intensive care unit (ICU) in Hangzhou, China, revealed that the prevalence of ST164_Pasteur_ had dramatically increased from <1.5% in 2019 to 49.2% in 2021 despite the predominance of GC2 isolates [91]. Interestingly, most of the ST164_Pasteur_ isolates from China harbour both *bla*_OXA-23_ and *bla*_NDM-1_ [90,91], but this is not the case for our seven ST164_Pasteur_ isolates. The first ST164_Pasteur_ isolate in our collection (AC15190) only appeared in 2015 (Data S1), and out of the total seven isolates, majority (*n*=5) were ST1418_Oxford_-KL47-OCL5, which was reflected in the analysis of global ST164_Pasteur_ isolates presented by Liu *et al.* [91]. Intriguingly, all our five ST164_Pasteur_-ST1418_Oxford_-KL47-OCL5 isolates were MDR and carbapenem resistant and harboured the *bla*_OXA-23_ and *bla*_CARB-5-like_ carbapenemases, whereas the remaining two ST164_Pasteur_ (which were ST234_Oxford_-KL196-OCL8 and ST2294_Oxford_-KL111-OCL8) isolates were non-MDR and carbapenem susceptible and only harboured the intrinsic *bla*_OXA-51-like_ gene (Fig. 4). Further vigilance and analysis of this GC11 lineage is warranted particularly due to its ability to displace the prevalent GC2 lineage in ICUs in a relatively short span of time [91]. Two novel Pasteur STs were found in the HSNZ *A. baumannii* isolates, i.e. ST2555_Pasteur_ (AC1839) and ST2575_Pasteur_ (AC1934), and these do not belong to any known clonal complexes (CCs). This suggests the continuous clonal evolution of *A. baumannii* in the hospital environment [92], and whether these clones will be successful in persisting in that environment will require further genomic surveillance.

Carbapenem resistance in *A. baumannii* is largely due to the horizontal acquisition of genes that encode carbapenem-hydrolysing enzymes of either Ambler class D (oxacillinases) or class B (MBL) [34]. For the Terengganu *A. baumannii* isolates, the class D OXA-23 (*n*=90/126; 71.4%) was predominant, while OXA-58 and the class B NDM-1 were found in only a few isolates. The *bla*_OXA-23_ gene is the most frequently identified acquired oxacillinase in *A. baumannii* [34], and other reports from Malaysia [84,85] and neighbouring countries such as Thailand, Myanmar and Vietnam also indicated likewise [40,86,89]. The class B MBL gene, *bla*_NDM-1_, was found in five *A. baumannii* isolates, and this includes *A. baumannii* AC1633 in which we previously reported its complete genome sequence by hybrid PacBio-Illumina assembly [20]. In AC1633, the *bla*_NDM-1_ gene was located in a 10 kb composite transposon Tn*125*, which, in turn, was found in a large ~170 kb plasmid designated as pAC1633-1. This plasmid also harboured *bla*_OXA-58_; the aminoglycoside resistance genes *aac(3)-IId*, *aph(3″)-Ib* and *aph(6)-Id*; the sulphonamide resistance gene *sul2*; and the macrolide resistance genes *msrE* and *mphE* [20]. A nearly identical plasmid was found in an MDR *Acinetobacter nosocomialis* AC1530 isolate from the same hospital a year earlier (2015), thereby hinting at the transmissibility of the plasmid although conventional conjugation assays performed failed to detect any transconjugants [20]. In this study, four other *A. baumannii* isolates were found carrying *bla*_NDM-1_, and two of these isolates (i.e. AC1839 which was ST2575_Pasteur_ from 2018 and AC2013 which was an ST257_Pasteur_ from 2020) were found to co-harbour the same suite of resistance genes as pAC1633-1. Two other *bla*_NDM-1_-positive isolates (AC1932 which was a ST1406_Pasteur_ from 2019 and AC2014 which was a ST142_Pasteur_ from 2020) harboured nearly all the resistance genes as pAC1633-1 except *bla*_OXA-58_. Since the genome sequences of these four isolates were obtained through the short-read DNBSeq platform, it was difficult to ascertain their plasmid content and architecture as the resistance genes involved were found in several separate contigs. Mapping of the pAC1633-1 sequences to the assembled contigs of these isolates appeared to indicate that a similar plasmid was present in these four isolates, but validation of this will require hybrid assembly with long-read sequencing data, which is currently being carried out. Nevertheless, the findings of *bla*_NDM-1_ (and to a lesser extent, *bla*_OXA-58_) and the other AMR genes that are carried on the pAC1633-1-type plasmid in *A. baumannii* isolates of different ST lineages strongly infer the likelihood of horizontal plasmid transfer occurring in these hospital isolates.

The carbenicillin-hydrolysing class A *β*-lactamase gene, *bla*_CARB-5-like_, was identified in 5/126 (3.97%) of the isolates with 3 variants detected, i.e. *bla*_CARB-5_, *bla*_CARB-16_ and *bla*_CARB-49_. CARB-16 is a single variant aa different from CARB-5 and was previously identified in one clinical *A. baumannii* isolate from Russia [93]. As stated above, all five Terengganu *A. baumannii bla*_CARB-5-like_-positive isolates belonged to ST164_Pasteur_/ST1418_Oxford_, and this result was in parallel with other Malaysian *A. baumannii bla*_CARB-5-like_-positive isolates that were previously reported [85]. Another class A ESBL gene, *bla*_TEM_, was found in 54/126 (42.86%) isolates, and this gene has been previously associated with resistance to third- and fourth-generation cephalosporins as well as carbapenems [94]. In this study, the *bla*_TEM_ gene was found exclusively in ST2_Pasteur_ isolates where 54/76 (71.1%) of the ST2_Pasteur_ *A. baumannii* isolates from HSNZ harboured this gene. The presence of the *bla*_TEM_ gene in ST2_Pasteur_ *A. baumannii* has been reported in previous studies [95,96].

Besides the various *β*-lactam resistance genes, other AMR genes and target site mutations were identified in the *A. baumannii* genomes, and these confer resistance to aminoglycosides, tetracyclines, sulphonamides, macrolides and fluoroquinolones. The two main mechanisms of resistance in *A. baumannii* against aminoglycosides are the enzymatic alteration of the aminoglycoside molecule by the aminoglycoside-modifying enzymes and 16S rRNA methylation by the *armA*-encoded enzyme [97,98]. Among the Terengganu *A. baumannii* clinical isolates, the most prevalent combinations of aminoglycoside resistance genes were *aph(3″)-Ib*, *aph(6)-Id*, *ant(3″)-llc* and *armA* (*n*=39/126; 31.0%) followed by *aph(3′)-Ia*, *aph(3″)-Ib*, *aph(6)-Id*, *ant(3″)-llc* and *armA* (*n*=26/126; 20.6%) with all the isolates displaying phenotypic resistance to aminoglycosides. More than half of the Terengganu *A. baumannii* isolates carried the *armA* gene (*n*=74/126; 58.7%), which has been reported to confer high-level resistance to aminoglycosides especially gentamicin, amikacin and tobramycin [99,100]. Majority of the *armA*-positive isolates belonged to ST2_Pasteur_ (*n*=68), and this was similarly reported in carbapenem-resistant *A. baumannii* isolates from Southern Thailand [86] and Singapore [101]. Sulphonamide resistance was mainly mediated by the *sul2* gene in the Terengganu *A. baumannii* isolates (*n*=61/126; 48.4%) with only one isolate harbouring *sul1*. The prevalence of the *sul2* gene has been reported in *A. baumannii* isolates from Thailand and China, whereas in Egypt, the *sul1* gene was reportedly more prevalent [86,102, 103]. The predominant tetracycline resistance genes in the Terengganu *A. baumannii* isolates were *tet(B)* (*n*=60/126) and *tet(39)* (*n*=10/126), and this was consistent with other studies [86,104, 105]. It should be noted that despite the presence of tetracycline resistance genes, none of the *A. baumannii* isolates were resistant to minocycline, leading to a zero-kappa value for minocycline when Cohen’s kappa coefficient analysis was used to evaluate the degree of concordance between bacterial isolates exhibiting the resistance phenotype and also possessing the corresponding resistance genotypes [106]. Minocycline was able to overcome the resistance afforded by *tet(B)* due possibly to minocycline possessing the strongest lipophilic ability and is the most potent agent in the tetracycline class [107]. Minocycline is also unaffected by the action of the AdeABC efflux pumps and has been shown to overcome many resistance mechanisms affecting other tetracyclines in *A. baumannii* [108,109]. Fluoroquinolone resistance was revealed to be due to mutations in the quinolone resistance-determining regions (QRDRs) of both the *gyrA* and *parC* genes. In this study, the coexistence of the *gyrA* subunit (S81L) and *parC* subunit (S84L, V104I and D105E) mutations was identified in majority (*n*=87/126; 69.1%) of the Terengganu *A. baumannii* clinical isolates. These mutations have also been observed in other *A. baumannii* isolates [110,111].

CRISPR-Cas systems in prokaryotes provide adaptive immunity against foreign DNA such as bacteriophages and plasmids [71].In a recent study on the prevalence of CRISPR-Cas systems in *Acinetobacter* spp. genomes, CRISPR-Cas, CRISPR arrays, or Cas proteins were identified in 150/6,824 (2.2 %) *A. baumannii* genomes with the majority of those being ST208_Oxford_ (11.5%), ST231_Oxford_ (8.5%) and ST540_Oxford_ (6.2%) [112]. Slightly higher prevalences were observed in this study (11.1%) and others [29,84]. Recent studies have indicated the involvement of CRISPR-Cas systems in modulating AMR in *A. baumannii* [71,75, 113]. Experiments conducted with *A. baumannii* AB43 showed that knocking out its entire type I-Fb CRISPR-Cas system [71] or just the *csy1* gene [75] led to a dramatic increase in resistance to multiple antibiotics even though AB43 did not harbour any acquired AMR genes. Transcriptomic analyses of the knocked-out *A. baumannii* strain indicated the increased expression of several multidrug efflux pumps with the quorum sensing autoinducer synthase gene, *abaI*, being shown as one of the targets for the type I-Fb CRISPR-Cas system [71]. Muzahid *et al.* [84] also reported that MDR hospital strains were less likely to harbour CRISPR-Cas systems as compared with drug-susceptible community isolates. However, our results appeared to be in contrast with these findings as of the 14 CRISPR-Cas-positive *A. baumannii* clinical isolates, 10 were MDR and only the remaining 4 were non-MDR. More work clearly needs to be done before a more conclusive finding could be made regarding the role (if any) that CRISPR-Cas systems play in AMR in *A. baumannii*.

## Conclusion

This study provides a snapshot of the AMR and genomic landscape of *A. baumannii* isolates obtained from a single tertiary hospital in the state of Terengganu, Malaysia, over a 10-year period (2011–2020). Majority of the *A. baumannii* isolates (*n*=97/126) were MDR, and all MDR isolates were carbapenem resistant. The GC2 lineage, especially ST2_Pasteur_, is clearly predominant, and although closely related, these isolates could be further subdivided based on their Oxford STs and KL types. The *bla*_OXA-23_-encoded acquired carbapenemase was predominant, and all MDR isolates also harboured various AMR genes and virulence-associated genes. Although the isolates analysed here were obtained from a single hospital, the WGS data obtained could be taken as a microcosm of the pathogen circulating in Malaysia over a 10-year period. This, in turn, will assist in the global surveillance of this highly resistant pathogen in the hopes of controlling its spread in the future.

## supplementary material

10.1099/mgen.0.001345 Uncited Supplementary Material 1.

10.1099/mgen.0.001345 Uncited Supplementary Material 2.

## Abbreviations

ADC: acinetobacter-derived cephalosporinase
AMR: antimicrobial resistance
ANI: average nucleotide identity
AST: antimicrobial susceptibility testing
CARD: comprehensive antibiotic resistance database
CCs: clonal complexes
CPS: capsular polysaccharide
CRISPR-Cas: clustered regularly interspaced short palindromic repeats-Cas
ESBL: extended-spectrum *β*-lactamase
GC: global clone
GC2: Global Clone 2
GC11: global clone 11
GFF: general feature format
HSNZ: Hospital Sultanah Nur Zahirah
IC: international clone
ICU: intensive care unit
KL: K locus
MBL: metallo-*β*-lactamase
MDR: multidrug resistant
ML: maximum likelihood
MLST: multilocus sequence typing
OCL: outer core locus
QRDRs: quinolone resistance-determining regions
RGI: resistance gene identifier
ST: sequence type
T6SS: type VI secretion system
VFDB: virulence factor database
WGS: whole genome sequencing
