# Supplementary material for: Whole-genome sequencing of Acinetobacter baumannii clinical isolates from a tertiary hospital in Terengganu, Malaysia (2011–2020), revealed the predominance of the Global Clone 2 lineage
Source: Microb Genom. 2025 Feb 5;11(2):001345. doi: 10.1099/mgen.0.001345 (PMC11798184; doi:10.1099/mgen.0.001345)
Supplement: Uncited Supplementary Material 1. [file mgen-11-01345-s001.pdf]

SUPPLEMENTARY MATERIAL_S1

Whole genome sequencing of *Acinetobacter baumannii* clinical isolates from a tertiary hospital in Terengganu, Malaysia (2011-2020) revealed the predominance of the Global Clone 2 lineage

Nurul Saidah Din[1], Farahiyah Mohd. Rani[1], Ahmed Ghazi Alattraqchi[1], Salwani Ismail[1], Nor Iza A. Rahman[1], David W. Cleary[2,3], Stuart C. Clarke[1,4,5,6,7]*, and Chew Chieng Yeo[1]*

[1]Centre for Research in Infectious Diseases and Biotechnology, Faculty of Medicine, Universiti Sultan Zainal Abidin, Kuala Terengganu, Malaysia;
[2]Department of Microbes, Infections and Microbiomes, School of Infection, Inflammation and Immunology, College of Medicine and Health, University of Birmingham, Birmingham, United Kingdom;
[3]Institute of Microbiology and Infection, University of Birmingham, Birmingham, United Kingdom;
[4]Faculty of Medicine and Institute for Life Sciences, University of Southampton, Southampton, United Kingdom;
[5]NIHR Southampton Biomedical Research Centre, University Hospital Southampton Foundation NHS Trust, Southampton, United Kingdom;
[6]Global Health Research Institute, University of Southampton, Southampton, United Kingdom;
[7]Institute for Research, Development and Innovation, International Medical University, Kuala Lumpur, Malaysia.

*__Corresponding authors:__
Stuart C. Clarke; S.C.Clarke@soton.ac.uk;
Chew Chieng Yeo; chewchieng@gmail.com

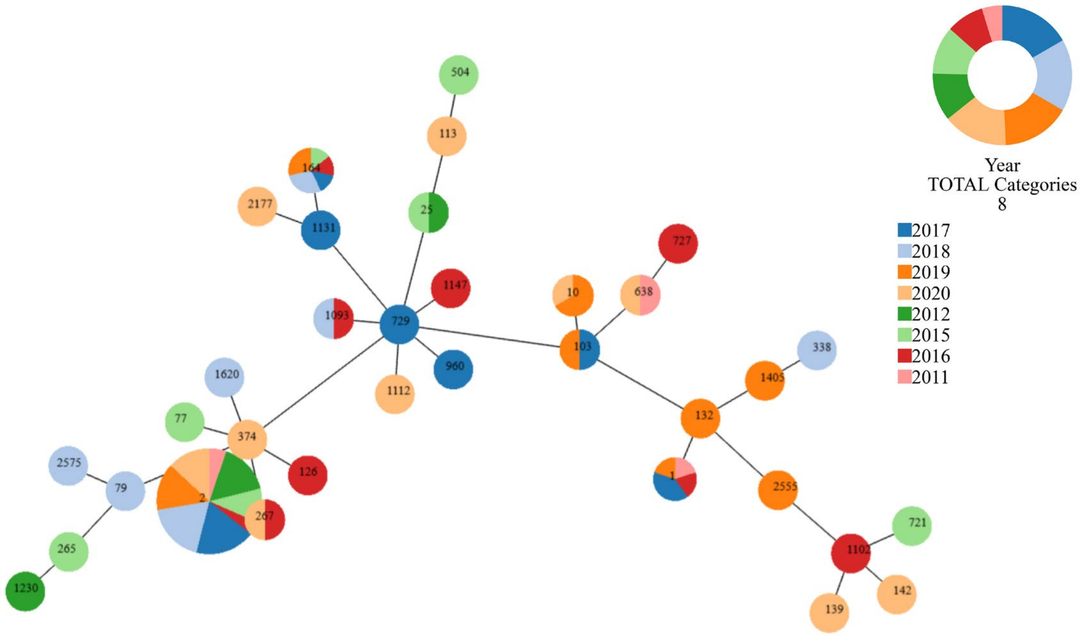

Supplementary Figure S1. Minimum spanning tree of the Pasteur STs identified in the 126 *A. baumannii* isolates in this study built with Phyloviz using the goeBURST algorithm (Ribeiro-Gonçalves et al., 2016). The Pasteur STs are labeled in each individual circle which are also coloured based on the year of isolation of each *A. baumannii* strain of that particular ST. The size of the circle correlated with the number of isolates for the particular ST.

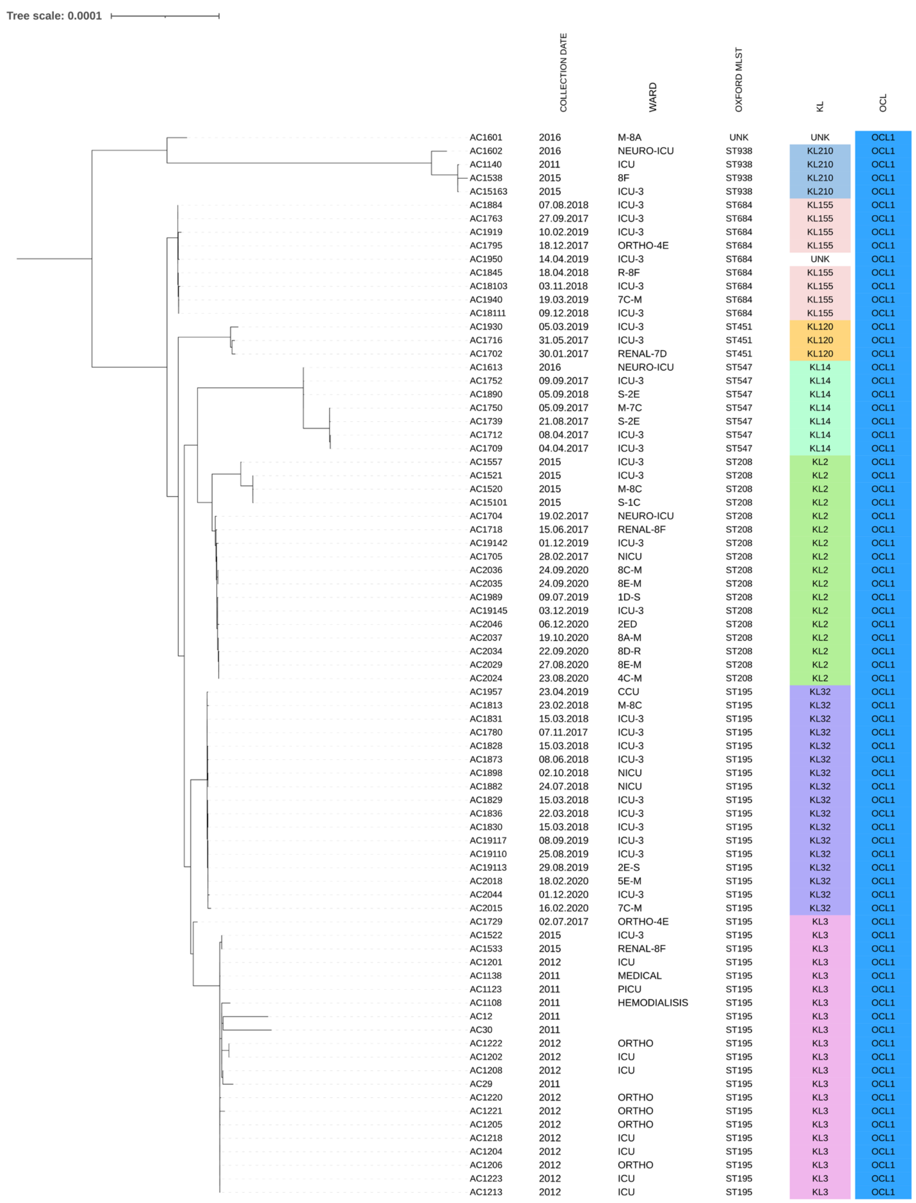

Supplementary Figure S2. The maximum-likelihood (ML) core genome phylogenetic tree of the 76 ST2$_{Pasteur}$ *A. baumannii* isolates and the ST2$_{Pasteur}$ reference strain from the same hospital (AC12, AC29 and AC30) was constructed using FastTree with 1000 bootstraps under the generalized time-reversible (GTR) model and was then visualized using iTOL (https://itol.embl.de/). The tree was annotated with their collection date, ward, Oxford MLST, the capsular polysaccharide locus (KL) and outer core biosynthesis locus (OCL) types. UNK indicates unknown.

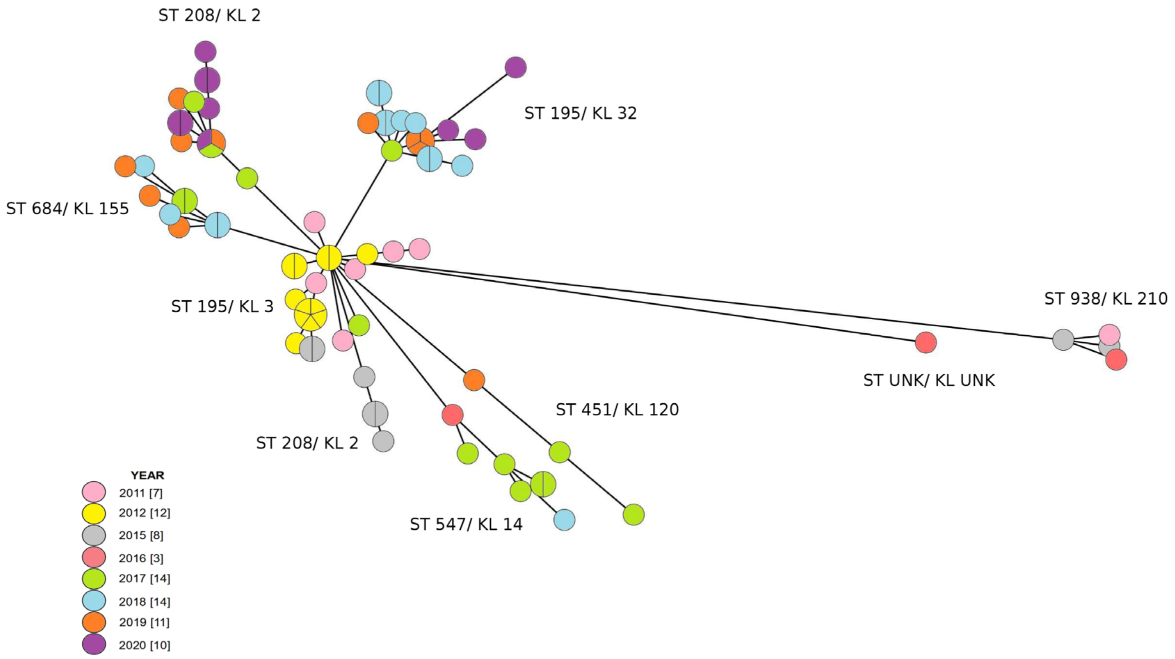

Supplementary Figure S3. Minimum spanning tree of the ST2$_{Pasteur}$ *A. baumannii* isolates in this study (*n* = 76) analysed by their Oxford STs and their KL loci and coloured according to their year of isolation. The tree was built using GrapeTree (Zhou et al., 2018; https://github.com/achtman-lab/GrapeTree).
